# Sex-Specific Changes to Brain Fatty Acids, Plasmalogen, and Plasma Endocannabinoids in Offspring Exposed to Maternal and Postnatal High-Linoleic-Acid Diets

**DOI:** 10.3390/ijms25147911

**Published:** 2024-07-19

**Authors:** Henry C. Ezechukwu, Luke J. Ney, Madeline A. Jarvis, Nirajan Shrestha, Olivia J. Holland, James S. M. Cuffe, Anthony V. Perkins, Suk-Yu Yau, Andrew J. McAinch, Deanne H. Hryciw

**Affiliations:** 1 School of Human Sciences, The University of Western Australia, Perth, WA 6009, Australia; henry.ezechukwu@research.uwa.edu.au; 2 School of Psychology and Counselling, Queensland University of Technology, Kelvin Grove, QLD 4059, Australia; luke.ney@qut.edu.au (L.J.N.); madeline.jarvis@connect.qut.edu.au (M.A.J.); 3 School of Pharmacy and Medical Science, Griffith University, Gold Coast, QLD 4222, Australia; nirajan.shrestha@griffithuni.edu.au (N.S.); o.holland@griffith.edu.au (O.J.H.); a.perkins@griffith.edu.au (A.V.P.); 4 School of Biomedical Sciences, The University of Queensland, Brisbane, QLD 4072, Australia; j.cuffe1@uq.edu.au; 5 School of Health, University of Sunshine Coast, Sippy Downs, QLD 4556, Australia; 6 Department of Rehabilitation Sciences, The Hong Kong Polytechnic University, Kowloon, Hong Kong; sonata.yau@polyu.edu.hk; 7 Mental Health Research Center, The Hong Kong Polytechnic University, Kowloon, Hong Kong; 8 Institute for Health and Sport, Victoria University, Melbourne, VIC 8001, Australia; andrew.mcainch@vu.edu.au; 9 Australian Institute for Musculoskeletal Science (AIMSS), Victoria University, St. Albans, VIC 3021, Australia; 10 School of Environment and Science, Griffith University, Nathan, QLD 4111, Australia; 11 Griffith Institute for Drug Discovery, Griffith University, Nathan, QLD 4111, Australia

**Keywords:** brain, fatty acids, linoleic acid, maternal diet, plasmalogen, endocannabinoids

## Abstract

Linoleic acid (LA) is required for neuronal development. We have previously demonstrated sex-specific changes in cardiovascular and hepatic function in rat offspring from mothers consuming a high-LA diet, with some effects associated with reduced LA concentration in the postnatal diet. At this time, the impact of a high-maternal-LA diet on offspring brain development and the potential for the postnatal diet to alter any adverse changes are unknown. Rat offspring from mothers fed low- (LLA) or high-LA (HLA) diets during pregnancy and lactation were weaned at postnatal day 25 (PN25) and fed LLA or HLA diets until sacrifice in adulthood (PN180). In the offspring’s brains, the postnatal HLA diet increased docosapentaenoate in males. The maternal HLA diet increased LA, arachidonate, docosapentaenoate, C18:0 dimethylacetal (DMA), C16:0 DMA, C16:0 DMA/C16:0, and C18:0 DMA/C18:0, but decreased eoicosenoate, nervoniate, lignocerate, and oleate in males. Maternal and postnatal HLA diets reduced oleate and vaccenate and had an interaction effect on myristate, palmitoleate, and eicosapentaenoate in males. In females, maternal HLA diet increased eicosadienoate. Postnatal HLA diet increased stearate and docosapentaenoate. Maternal and postnatal HLA diets had an interaction effect on oleate, arachidate, and docosahexaenoic acid (DHA)/omega (n)-6 docosapentaenoic acid (DPA) in females. Postnatal HLA diet decreased DHA/n-6 DPA in males and females. Postnatal HLA diet increased plasma endocannabinoids (arachidonoyl ethanolamide and 2-arachidonoyl glycerol), as well as other N-acyl ethanolamides and testosterone. HLA diet alters brain fatty acids, plasma endocannabinoids, and plasmalogen concentrations in a development-specific and sex-specific manner.

## 1. Introduction

Linoleic acid (LA) is an essential omega (n)-6 polyunsaturated fatty acid (PUFA) that is required for normal cellular function and is thus critical for fetal health and development [1]. LA overconsumption has been associated with an increased risk of obesity [2]. Furthermore, the overconsumption of n-6 PUFAs during pregnancy and lactation predisposes the developing fetus and offspring to adverse programming outcomes [3]. We have previously shown that a diet high in LA promotes maternal inflammation during pregnancy in rats [4], as well as cardiovascular [5] and hepatic [6] changes in offspring during adolescence, and hepatic changes in adulthood [7]. There are multiple proposed mechanisms linking maternal overconsumption of n-6 PUFAs to adverse offspring outcomes, including maternal inflammation [8], and we have previously demonstrated altered accumulation of specific fatty acids in tissues such as the brain, or altered endocannabinoid signaling. Similarly, the pathological outcomes seen in offspring prenatally exposed to n-6 PUFAs may be due to programmed changes in circulating hormones or inflammatory markers. In mice, elevated maternal inflammation during pregnancy negatively impacts neurodevelopment [9]. To add to this, we have demonstrated sex-specific changes in behavior in offspring exposed to a maternal high-LA diet during pregnancy and early life [10]. Others have shown that high maternal LA intake induces neurological dysfunction such as ataxia and encephalomalacia [1]. The brain tissue is rich with diverse lipid populations [11]. Changes in lipid composition have been widely reported to be associated with neurological defects such as Alzheimer’s disease [12,13], Zellweger syndrome [12,14], and Niemann–Pick syndrome [12,15]. LA is also a precursor to oxidized products known as ‘oxidized linoleic acid metabolites’ (OXLAMs) [1]. OXLAMs are lipid mediators in the brain and are known to regulate pain [16] and inflammation [17]. 

Plasmalogen is a class of glycerophospholipid characterized by the presence of a vinyl-ether bond at the C-1 position [17] and enriched PUFAs, including docosahexaenoic acid (DHA; C22:6 n-3) or arachidonic acid (AA; C20:4 n-6), at C-2 of the glycerol moiety [18]. Imbalances in PUFA concentrations in the brain have the capacity to program fetal neuronal development [19]. Plasmalogens are glycerophospholipids that are major components of mammalian cellular membranes, particularly in the brain [20]. Plasmalogen synthesis participates in key physiological processes including cholesterol hemostasis [21], scavenging reactive free radicals, signal transduction, ion transport, storage for PUFAs and lipid mediators [12], and modulation of myelin sheath formation in the brain, particularly during development [22]. Brites et al. [22] reported that plasmalogens prevent inflammatory demyelination and axonopathy induced by long-chain fatty acids. Monounsaturated fatty acids (MUFAs), PUFAs, and DHA are abundant in the brain [23]. Deficiency of plasmalogen biosynthesis is associated with lipid storage diseases and neurological defects including Gaucher diseases [24] and Alzheimer’s disease [18,23]. 

The endogenous cannabinoid (endocannabinoid) system is a lipid signaling system [25] that consists of receptors located within both the brain and the peripheral nervous system [26]. The two major endogenous cannabinoid receptor ligands, arachidonyl ethanolamide (AEA) and 2-arachidonoyl glycerol (2-AG), are degraded by monoacylglycerol lipase (MAGL) and fatty acid amide hydrolase (FAAH) to produce arachidonic acid (AA) [27]. Metabolism of LA also produces AA, which can be catabolized into different metabolites through cyclooxygenase (COX), lipoxygenase (LOX), and cytochrome P450 (CYP-450) into pro-inflammatory prostaglandins [28]. The endocannabinoid system is critical to fundamental synaptic processes, including long-term potentiation and depression [29], implying a significant role in early brain and lifespan development, as well as learning, memory, and general brain development [30]. Given their role as secondary messengers, endocannabinoids have been demonstrated to mediate the actions of other neurotransmitter systems (e.g., dopamine, glucocorticoids, and serotonin) [31], further reinforcing the fundamental importance of understanding endocannabinoid signaling in healthy development. Other endocannabinoid-like compounds such as oleoyl ethanolamide (OEA), palmitoyl ethanolamide (PEA), stearoyl ethanolamide (SEA), and linoleoyl ethanolamide (LEA) are also AA metabolites and are considered to be part of the expanded endocannabinoid system, but they do not interact directly with cannabinoid receptors [25].

A mother’s diet during pregnancy programs disease risk in her offspring in later life [32]. An adverse maternal environment during pregnancy and lactation can sometimes be reversed with modifications to the offspring’s diet and lifestyle during critical, postnatal periods of development. For example, in a study by Raipuria et al., the adverse effects of an obesogenic maternal diet on offspring’s metabolic health could be reversed by postnatal exercise [33]. However, other studies have demonstrated that the impacts of adverse prenatal diets are permanent and are unable to be reversed with dietary intervention in offspring. Campodonico-Burnett et al. demonstrated that postnatal consumption of control or low-fat diets did not reverse the adverse effects on metabolism in rats following exposure to an obesogenic maternal environment in utero [34]. Furthermore, it is possible that secondary exposure to a poor diet in postnatal life can exacerbate poor health outcomes [35]. The permanency of programmed changes to physiology following prenatal n-6 PUFA exposure likely depends on the mechanism of action. It is possible that n-6 PUFA exposure forever influences the fatty acid composition of the brain as it is being formed, but that lipid turnover in the brain is limited in postnatal life such that postnatal lipid exposure does not impact long-term outcomes to the same extent. Therefore, the potential for the reversal or exacerbation of any adverse perturbations in offspring exposed to a poor uterine environment should be investigated. 

The effects of prolonged exposure to different levels of LA before pregnancy, during gestation, and during lactation and/or after weaning on the fatty acid concentrations and plasmalogen contents in offspring’s brains and the endocannabinoids in their plasma are yet to be investigated. In this study, we aim to investigate the effects of maternal and postnatal HLA diet intake on the fatty acid composition and plasmalogen concentrations in the brain, and endocannabinoids in plasma, of adult rodent offspring. 

## 2. Results

### 2.1. Maternal and Postnatal Consumption of HLA Diet Affects the Brain’s Saturated Fatty Acid, Monounsaturated Fatty Acid, and Polyunsaturated Fatty Acid Composition in Adult Offspring in a Sex-Dependent Manner

In the brains of male offspring, maternal HLA diet increased C18:0 dimethylacetal (DMA) (*p* = 0.0022), C16:0 DM (*p* = 0.0001), linoleate (C18:2n6; *p* = 0.0017), and arachidonate (AA; C20:4n6; *p* < 0.001) (Table 1, *n* = 6–8). In the brains of male offspring, maternal HLA diet decreased lignocerate (C24:0; *p* = 0.0371), eicosenoate (C20:1n9; *p* = 0.0068), and nervoniate (C24:1n9; *p* = 0.0324) (Table 1, *n* = 6–8). In the brains of male offspring, postnatal HLA diet increased docosapentaenoate (DPA; C22:5n6; *p* < 0.0001) (Table 1, *n* = 6–8).

In the brains of male offspring, both maternal and postnatal HLA diets decreased oleate (C18:1n9) (*p* = 0.0042 and *p* = 0.0008, respectively) and vaccenate (C18:1) (*p* = 0.001 and *p* = 0.0251, respectively) (Table 1, *n* = 6–8). There was an interaction effect for maternal and postnatal HLA diets on myristate (C14:0; *p* = 0.0095), palmitoleate (C16:1n7; *p* = 0.0095), and EPA C20:5n3 (*p* = 0.0095) in the brains of male offspring (Table 1, *n* = 6–8). 

In the brains of female offspring, postnatal HLA diet increased stearate (C18:0; *p* = 0.0409) and docosapentaenoate (DPA; C22:5n6; *p* < 0.0001) (Table 2, *n* = 6–8). In the brains of female offspring, maternal HLA diet increased eicosadienoate C20:2n6 (*p* = 0.0083) (Table 2, *n* = 6–8). Both maternal and postnatal HLA diets had an interaction effect on the increased oleate (C18:1n9; *p* = 0.0221) and arachidate (C20:0; *p* = 0.003) in the brains of female offspring (Table 2, *n* = 6–8).

### 2.2. Maternal and Postnatal Consumption of an HLA Diet Affects Brain AA/DHA, DHA/n-3 DPA, and DHA/n-6 DPA in a Sex-Dependent Manner

**Figure 1:**
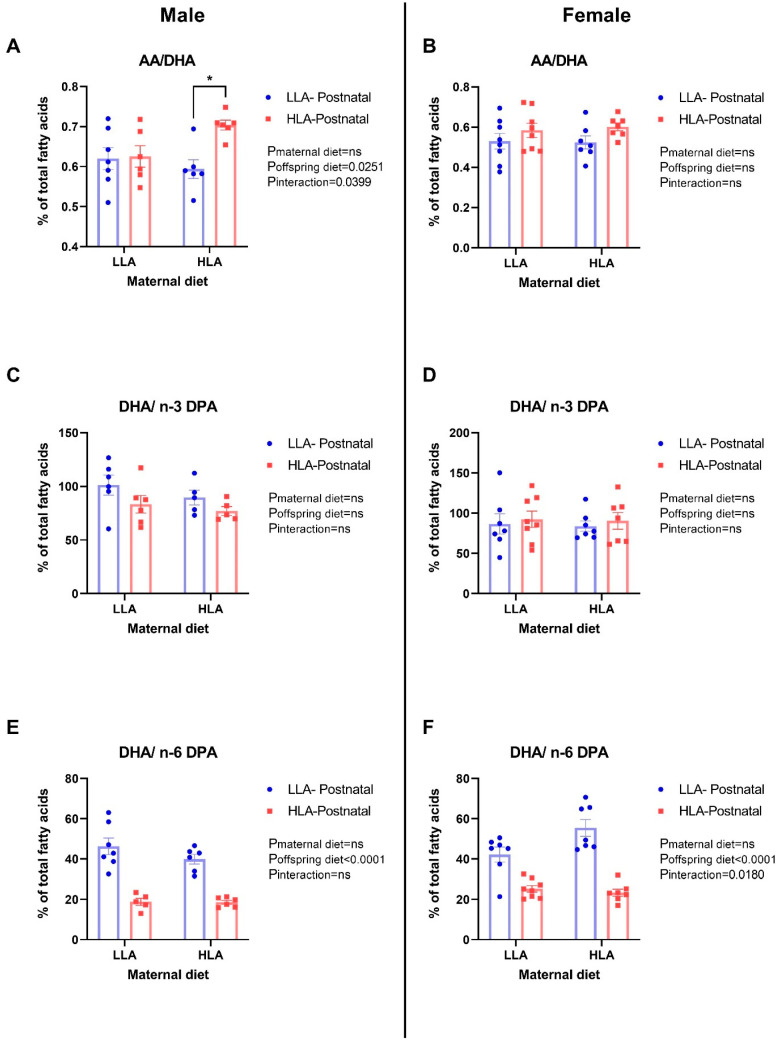
Effects of maternal and postnatal diets high in linoleic acid on arachidonic acid/docosahexaenoic acid (AA/DHA) (**A**,**B**), docosahexaenoic acid/n-3 docosapentaenoic acid (DHA/n-3 DPA) (**C**,**D**), and docosahexaenoic acid/n-6 docosapentaenoic acid (DHA/n-6 DPA) (**E**,**F**) in the brains of male (**A**,**C**,**E**) and female (**B**,**D**,**F**) offspring. Two-way ANOVA was performed with maternal diet and postnatal diet as two factors. LLA: low linoleic acid; HLA: high linoleic acid. Data are expressed as the mean ± standard error of the mean (SEM); ns = not significant; *n* = 6–8, * *p* < 0.05.

Postnatal HLA diet significantly increased AA/DHA (*p* = 0.0251) in male offspring (Figure 1, *n* = 6–8). Both maternal and postnatal HLA diets had an interaction effect on the increased AA/DHA (*p* = 0.0399) in male offspring (Figure 1). Postnatal HLA diet decreased DHA/n-6 DPA (*p* < 0.0001) in both male and female adult offspring (Figure 1, *n* = 6–8). Moreover, maternal and postnatal HLA diets had an interaction effect on the decreasing DHA/n-6 DPA (*p* = 0.018) in female adult offspring (Figure 1, *n* = 6–8). There were no differences in DHA/n-3 DPA among all groups in both sexes (Figure 1, *n* = 6–8).

### 2.3. Maternal HLA Diet Increases Brain Plasmalogen (C16:0 DMA/C16:0 and C18:0 DMA/C18:0) in Male Offspring

**Figure 2:**
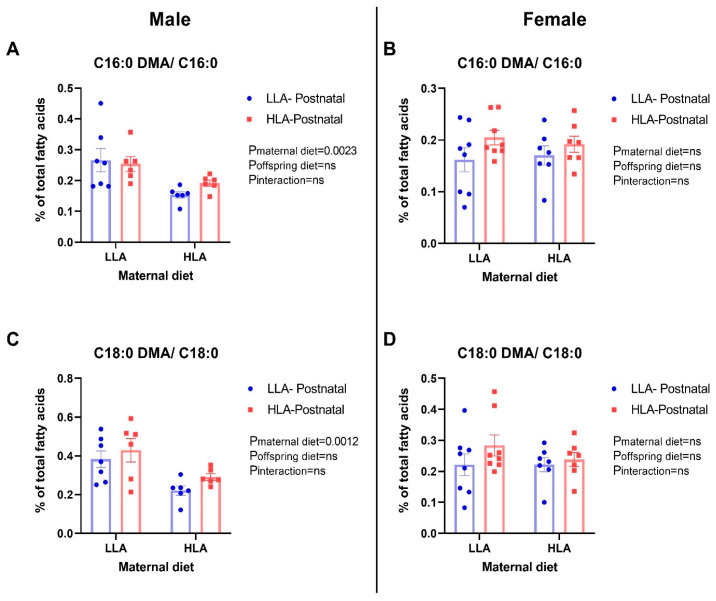
Effects of maternal and postnatal diets high in linoleic acid on C16:0 dimethylacetal (DMA)/C16:0 (**A**,**B**) and C18:0 dimethylacetal (DMA)/C18:0 (**C**,**D**) in the brains of male (**A**,**C**) and female (**B**,**D**) offspring. Two-way ANOVA was performed with maternal diet and postnatal diet as two factors. LLA: low linoleic acid; HLA: high linoleic acid. Data are expressed as the mean ± standard error of the mean (SEM); ns = not significant, *n* = 6–8.

We further compared C16:0 or C18:0 to their respective DMA. Maternal HLA diet increased both C16:0 DMA/C16:0 (*p* = 0.0023; Figure 2, *n* = 6–8) and C18:0 DMA/C18:0 (*p* = 0.0012) in adult male offspring (Figure 2, *n* = 6–8). There were no changes in brain plasmalogen in female offspring (Figure 2, *n* = 6–8). 

### 2.4. Maternal and Postnatal HLA Diets Alter Plasma Endocannabinoids in Adult Offspring, in a Sex-Specific Manner

**Figure 3:**
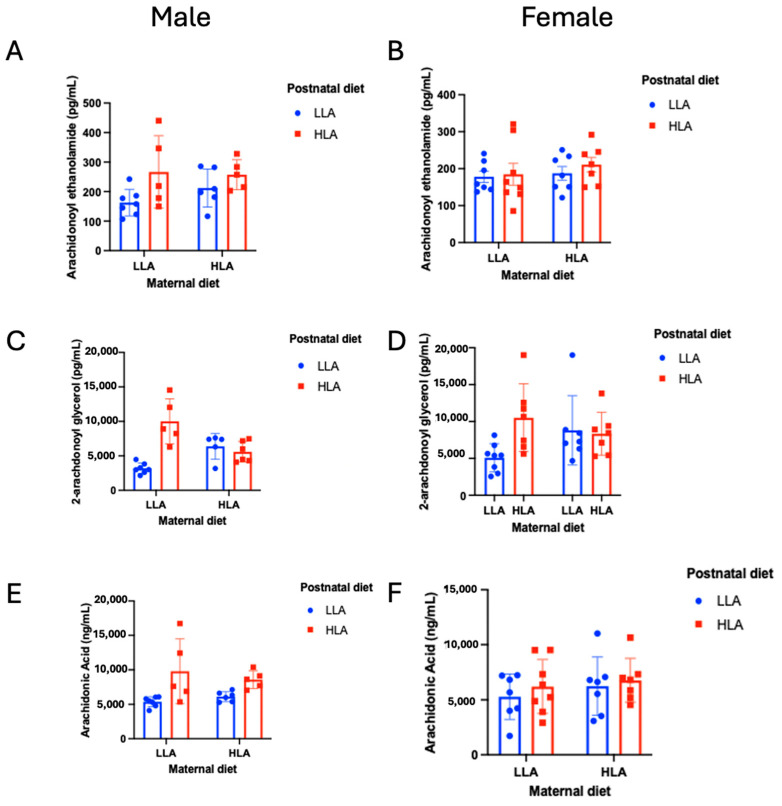
Effects of maternal and postnatal diets high in linoleic acid on plasma arachidonoyl ethanolamide (**A**,**B**), 2-arachidonoyl glycerol (**C**,**D**), and arachidonic acid (**E**,**F**) in male (**A**,**C**,**E**) and female (**B**,**D**,**F**) offspring. Three-way ANOVA was performed with maternal diet, sex, and postnatal diet as three factors. LLA: low linoleic acid; HLA: high linoleic acid. Data are expressed as the mean ± standard error of the mean (SEM); ns = not significant; *n* = 6–8.

Plasma endocannabinoids (AEA and 2-AG) were altered by both maternal and postnatal diets (Figure 3, *n* = 6–8), irrespective of sex. For AEA, there was a significant effect of postnatal diet (*p* = 0.031), with higher AEA in the HLA diet group. The interaction between sex and postnatal diet did not reach significance (*p* = 0.083), and while plasma AEA seemed higher in males overall compared to females, this was not significant (*p* = 0.072). For 2-AG, there was a significant effect of sex (*p* = 0.043), postnatal diet (*p* < 0.001), and maternal × postnatal diet (*p* = 0.003). Females had significantly higher plasma 2-AG compared to males, and overall postnatal HLA diet resulted in higher 2-AG, but only when the maternal diet was LLA. 

AA was significantly higher in males compared to females (*p* = 0.042), as well as in postnatal HLA compared to LLA diets (*p* = 0.002). There was also a significant sex × postnatal diet interaction for AA (*p* = 0.044), with significantly higher AA in males with an HLA compared to an LLA postnatal diet (*p* = 0.002), but a smaller, non-significant increase in AA in females fed a postnatal HLA rather than LLA diet (*p* = 0.382). No other effects for AEA, AA, or 2-AG were significant (*n* = 6–8).

### 2.5. Effects of Maternal and Postnatal HLA Diets on Plasma N-acyl Ethanolamide in Adult Offspring

**Figure 4:**
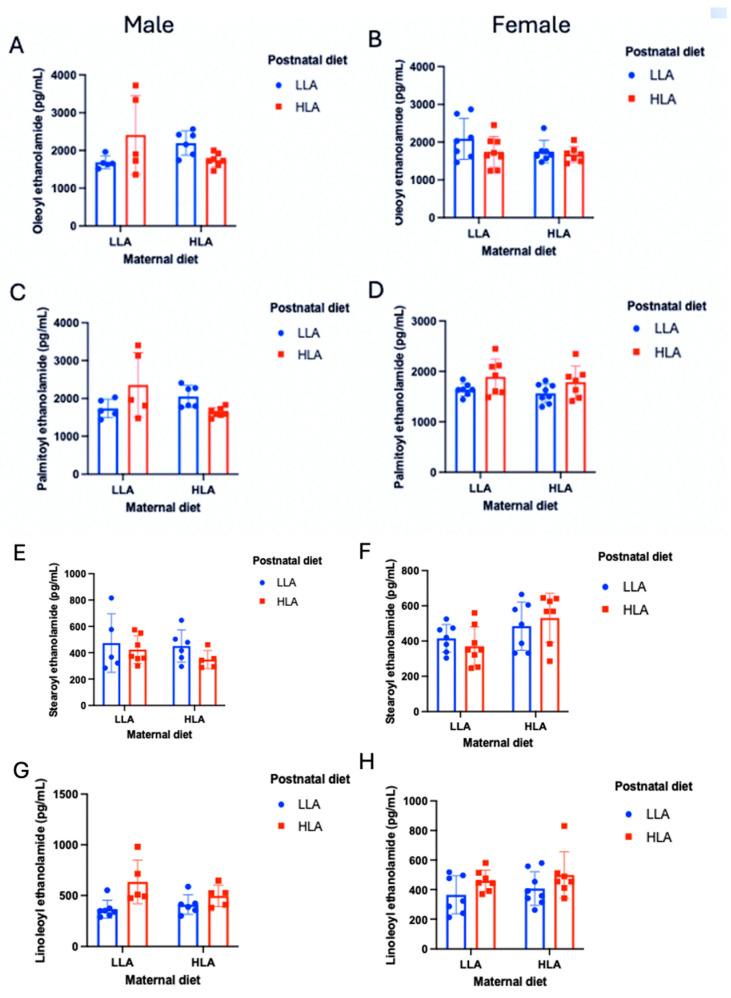
Effects of maternal and postnatal HLA diets on plasma oleoyl ethanolamide (**A**,**B**), palmitoyl ethanolamide (**C**,**D**), stearoyl ethanolamide (**E**,**F**), and linoleoyl ethanolamide (**G**,**H**) in male (**A**,**C**,**E**,**G**) and female (**B**,**D**,**F**,**H**) offspring. Three-way ANOVA was performed with maternal diet, sex, and postnatal diet as three factors. LLA: low linoleic acid; HLA: high linoleic acid. Data are expressed as the mean ± standard error of the mean (SEM); *n* = 6–8.

Plasma N-acyl ethanolamides (OEA, PEA, SEA, LEA) were compared between maternal and postnatal diets (Figure 4, *n* = 6–8) and were again found to differ as a function of diet, but also as a function of sex in some cases. The only significant effect for OEA revealed a sex × maternal × postnatal diet interaction (*p* = 0.007). This interaction effect suggested that a postnatal HLA diet was only associated with increased plasma OEA in males that had maternal LLA diets (*p* = 0.016). This interaction was not found in females (*p* = 0.351). A similar effect was found for PEA, where the sex × maternal × postnatal diet interaction was significant (*p* = 0.014), with increased plasma PEA in males fed HLA postnatal diets, but only when they had maternal LLA diets. This test also found that PEA was higher overall in males (*p* = 0.028), and the maternal × postnatal diet interaction was significant (*p* = 0.009), though this was superseded by the three-way interaction that revealed that this interaction occurred only in males (*p* = 0.011) but not in females (*p* = 0.865). No effects of SEA were significant, including the sex × postnatal diet interaction, which trended towards, but did not reach, significance (*p* = 0.053). Finally, plasma LEA was significantly higher in the postnatal HLA group (*p* < 0.001), though this was not moderated by sex (*p* = 0.240), and no other significant effects were observed.

### 2.6. Effects of Maternal and Postnatal HLA Diets on Plasma Steroid Hormones in Adult Offspring

**Figure 5:**
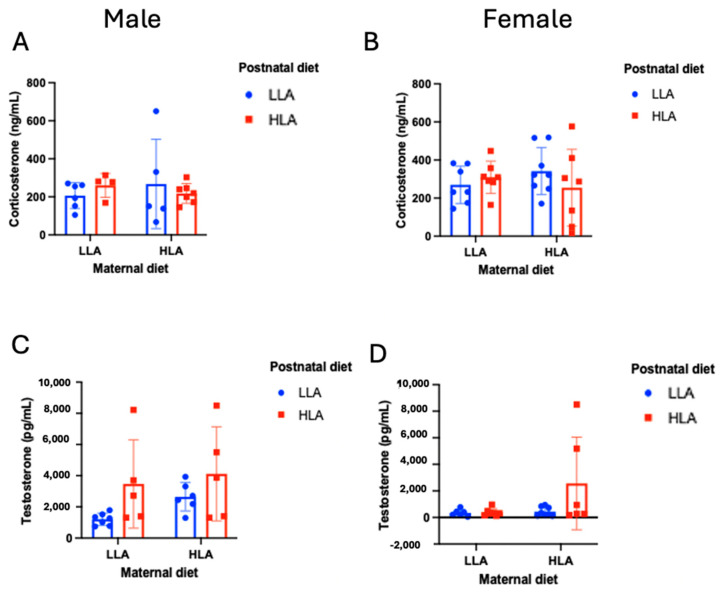
Effects of maternal and postnatal diets high in linoleic acid on plasma corticosterone (**A**,**B**) and testosterone (**C**,**D**) in male (**A**,**C**) and female (**B**,**D**) offspring. Three-way ANOVA was performed with maternal diet, sex, and postnatal diet as three factors. LLA: low linoleic acid; HLA: high linoleic acid. Data are expressed as the mean ± standard error of the mean (SEM); ns = not significant; *n* = 6–8.

As n-6 PUFA exposure may mediate effects on offspring’s physiology through indirect actions on their hormones, we measured corticosterone and testosterone concentrations. While no effects were observed for corticosterone, there was a significant effect of LA diet on plasma testosterone (Figure 5, *n* = 6–8). There was a significant main effect of testosterone (*p* < 0.001), with males having higher testosterone than females. There were also significant main effects of maternal diet (*p* = 0.040) and postnatal diet (*p* = 0.007), such that significantly higher testosterone was observed in maternal and postnatal HLA diet conditions. Although these effects did not occur in females with LLA postnatal diets, the three-way interaction did not reach significance (Figure 5, interaction term *p* = 0.159, *n* = 6–8), and all other interactions featuring the factor of sex in the ANOVA were non-significant.

## 3. Discussion

The brain is rich in lipids, and dietary fatty acids in the brain can impact brain function and behavior [36]. A significant novel outcome from this study is that consumption of a high-LA diet prior to and during pregnancy/weaning altered the brain’s fatty acid concentrations in addition to C16:0 and C18:0 DMA concentrations in offspring. Changes in plasmalogen concentrations only occurred in male offspring. To the best of our knowledge, this is the first study to investigate the effects of maternal and postnatal HLA diets on offspring brain fatty acid composition and brain plasmalogen. In this study, we provide new evidence that brain plasmalogen is affected by a maternal diet high in LA, and not a postnatal diet, in a sex-specific manner. Furthermore, the ratio of DHA/n-6 DPA is reduced by postnatal high-LA diets in both sexes, with an interaction effect in females. There was greater diversity in the concentration of fatty acids in the brains of males compared with females, suggesting that the developing male brain is more susceptible to elevated LA concentrations. Finally, we found evidence that high-LA diet affected endocannabinoids as well as plasma testosterone, often in a sex-specific manner, with stronger effects in males compared to females. These effects were most prominent with the postnatal diet, though often only when the maternal diet was low in LA, suggesting a potential restorative effect and an opportunity for postnatal treatment.

The consumption of LA has increased 20-fold in Western countries in recent decades among people within reproductive age [37]. The increasing consumption of LA during pregnancy is linked to adverse outcomes in offspring neurodevelopment [1]. The ratio of specific fatty acids is an important indicator of neuronal dysfunction [37]. Mechanistically, previous research has demonstrated that a high AA/DHA ratio induces triglyceride accumulation, increases oxidative stress, and disrupts mitochondrial functions [38]. DPA is metabolized by the enzymes fatty acid elongase 2 (ELOVL2) and fatty acid desaturase 1 (FADS1) [39]. The reduction in DHA/n-6 DPA ratio in response to an HLA diet was likely due to the elevated n-6 DPA, which is similar to the findings of a previous study [40]. Interestingly, in the sera of pregnant women, the reduction in DHA/n-6 DPA has been linked to depression [41]. Furthermore, in rodents, an increase in neuronal n-6 DPA leads to deficits in learning and memory assessed through the Morris water maze task [42]. Mechanistically, this may be due to FADS1/2, with polymorphisms in FADS1/2 linked to neurobehavioral disorders [43].

During pregnancy, the fetus depends on an adequate supply of maternal AA and DHA for optimal brain growth, obtained from metabolism of LA and ALA, respectively [44]. We have previously shown that there is a significant increase in the concentrations of AA and LA in male offspring born to mothers fed with a diet high in LA [4], albeit at embryonic day 20. In the current study, we did not observe significant effects of postnatal HLA on brain AA, LA, and DHA composition in either of the sexes in adulthood (PN180). However, maternal HLA diet significantly altered AA and LA concentrations in the brains of male offspring, which may have been set up by the increased AA and LA in plasma at E20. As AA promotes inflammation [45], this suggests that a maternal diet high in LA may impact neurodevelopment in the male offspring. 

The n-3 long-chain fatty acids are crucial in the development of neural tissues [46], and they are metabolized to DHA via a series of biochemical pathways [47]. Several studies have linked the supplementation of diet with n-3 fatty acids during pregnancy and lactation with increased mental IQ in children later in life [44,48]. Mechanistically, DHA and n-3 DPA concentrations in the diet have been associated with reduced cardiovascular disease incidence compared to n-6 DPA [49]. Data from 41 studies in different countries show that low DHA in maternal milk is associated with an increased risk of postpartum depression [50,51]. Similarly, offspring born to mothers with low DHA in their breast milk have an increased risk of depression [51]. Further studies have shown that prolonged feeding with a diet low in n-3 PUFAs decreases DHA in the brains of either male or female adult mice [52,53]. Despite no effect on DHA concentrations in this study, there was a decrease in DHA/n-6 DPA in both sexes due to postnatal high-LA diet intake, along with an interaction effect in female offspring. This suggests that there was a reduction in brain DHA, which may negatively impact neurodevelopment and function. 

In Western cultures, an elevated n-6 dietary intake is often associated with a high dietary fat intake. Several reports have shown that the intake of a high-fat diet alters specific SFA, MUFA, and PUFA profiles, including increased C18:0 DMA in the retina [54]. Similarly, our results show significant alterations in SFA, MUFA, and PUFA concentrations in the brains of offspring due to maternal consumption of an HLA diet, with more significant changes observed in the male offspring. This suggests that, in part, the consumption of a high-fat diet may alter SFA, MUFA, and PUFA concentrations due to LA. 

The transmethylation of SFA C18:0 and C16:0 products (DMA 18:0 and DMA C16:0) was increased in male adult offspring born to mothers fed with a diet high in LA. This may indicate that there was altered brain lipid metabolism in male offspring. Plasmalogens are a class of phospholipids that constitute about 18% of total phospholipids and are abundant in neurons, as well as skeletal and cardiac muscles [24]. Plasmalogens play a crucial role in protecting brain cells from reactive oxidative species damage and serve as a major source of AA. DMA is currently used to reflect plasmalogen concentrations in central and peripheral tissues [24]. Decreased plasmalogen concentrations are associated with several pathologies, such as spingolipidoses and peroxisomal disorders [18,24]. In our study, we observed that maternal HLA diet increased C16:0 DMA/C16:0 and C18:0 DMA/C18:0 in the brains of male offspring. This may lead to neural damage in male offspring, which may in part be due to an inflammatory assault contributed by AA mediators, including n-6 DPA. Conversely, the female offspring showed no significant alterations in brain C16:0 DMA/C16:0 and C18:0 DMA/C18:0, even when exposed to postnatal diet high in LA, suggesting that the brain plasmalogen was not significantly affected in female offspring exposed to an HLA diet. Sex differences exist due to the capacity of ovarian hormones, i.e., estrogen, to modulate neuronal lipid metabolism [55]. The reduction in plasmalogen in males may indicate that estrogen buffers against the negative impact of a maternal high-LA diet. Future research should focus on elucidating the molecular mechanisms involved in plasmalogen’s modulation of neurofunction and the behavioral consequences in offspring exposed to an elevated-LA diet.

Endocannabinoids are metabolite products of dietary essential PUFAs and are crucial to early brain development [56]. The current study demonstrated that AEA, 2-AG, AA, OEA, PEA, and LEA were significantly higher in postnatal high-LA diets compared to postnatal low-LA diets. For 2-AG, OEA, and PEA, these effects were only significant when the maternal diet was low in LA, suggesting a restorative effect and a potential avenue for future treatments. However, for OEA and PEA, this only occurred in males, with no changes in OEA or PEA in males with a postnatal HLA diet. Further reinforcing the sex differences found in these data, AA increased as a function of postnatal LA diet, but only in males, and AEA, 2-AG, and AA all showed evidence of overall sexual dimorphism in terms of plasma concentrations. Sex differences in overall endocannabinoid tone have previously been identified in blood, hair, and saliva samples [57], and the endocannabinoid system shows stark evidence of sexual dimorphism in terms of endocannabinoid activity in health and disease [58,59,60,61]. The underlying mechanisms for this may be in part due to the sexually dimorphic expression of enzymes responsible for the metabolism of endocannabinoids, namely, MAGL and FAAH, with females having a higher amygdala expression of MAGL and FAAH in rats [62]. These findings were reinforced by the significant effects of LA diet on testosterone in the current study. Specifically, higher testosterone was found in males exposed to postnatal HLA diets. These findings should be further investigated in future research, as they may hold important information as to why sexual differences in endocannabinoid signaling occur.

## 4. Materials and Methods

### 4.1. Experimental Animal Model and Diet

Ethical approval was granted by the Griffith University Animal Ethics Committee (NSC/01/17/AEC). Female Wistar Kyoto rats (8 weeks of age) were purchased from the Australian Resource Centre (ARC, Perth, WA, Australia) and housed in accordance with the Australian Code of Practice for Care and Use of Animals for Scientific Purpose. 

Female rats consumed either a diet with low LA (LLA) or high LA (HLA) for 10 weeks. Females were then mated with a control male (consuming standard rat chow). Following mating, the female rat continued to consume the same diet as prior to pregnancy during pregnancy and until weaning. Offspring were weaned at postnatal day (PN) 25 and fed with either an LLA or HLA diet. This gave rise to 4 groups of offspring (LLA maternal–LLA PN; LLA maternal–HLA PN, HLA maternal–LLA PN, and HLA maternal–HLA PN). At PN180, the offspring were euthanatized by intraperitoneal injection with sodium pentobarbital (60 mg/kg) [7]. Their brains were dissected and weighed, and then snap-frozen at −80 °C until use. Only one male and one female from each litter were used for each analysis; n values represent individual offspring from separate litters.

### 4.2. Rodent Brain Fatty Acid Analysis 

Fatty acid composition was quantified by Cardinal Bioresearch as described previously [63]. Briefly, the brain fatty acid composition was analyzed by gas chromatography (GC) with flame ionization detection. The tissue was transferred to a screw-cap glass vial containing the methylation reagent (14% boron trifluoride, toluene, methanol; 35:30:35 *v*/*v*/*v*; Sigma-Aldrich, St. Louis, MO, USA). The tissue was sonicated for 5 min, and the sample was vortexed and then incubated a hot bath at 100 °C for 45 min. Hexane (EMD Chemicals, Gibbstown, NJ, USA) and HPLC-grade water were added following cooling, and separate layers were generated through centrifugation. An aliquot of the hexane layer was transferred to a GC vial. GC was carried out using a GC-2030 Gas Chromatograph (Shimadzu Corporation, Columbia, MD, USA) equipped with an SP-2560, 100 m fused silica capillary column (0.25 mm internal diameter, 0.2 um film thickness; Restek, Bellefonte, PA, USA). A standard mixture of fatty acids (NuCheck Prep, Elysian, MN, USA) was used to identify individual fatty acids. Fatty acid composition was expressed as a percentage of the total identified fatty acids. 

### 4.3. Plasma Endocannabinoid Analysis

Endocannabinoid analysis was performed as previously described [64,65], with some modifications. Briefly, we performed stable isotope dilution using liquid chromatography paired with tandem mass spectrometry (LC-MS/MS). Isotopically labeled standards d4-AEA, d5-2-AG, d4-OEA, d4-PEA, d4-hydrocortisone, d8-arachidonic acid, and d3-testosterone were added to 200 µL of each rat plasma sample. Then, 1 mL of 50:50 cyclohexane/ethyl acetate extraction solution was added to each sample, after which the samples were vortexed, centrifuged, and the resulting supernatant concentrated to a final 15 µL acetonitrile solution, of which 5 µL was injected into the LC-MS/MS system. The LC-MS/MS system included a Nexera X2 UHPLC (Shimadzu, Sydney, Australia) binary pump system coupled with a Sciex QTRAP 6500. (Sciex, Mt Waverley, Australia) Mobile phase A consisted of 0.2 mM ammonium fluoride, and mobile phase B was 100% acetonitrile. Plasma AEA, 2-AG, OEA, PEA, SEA, LEA, AA, corticosterone, and testosterone were calculated in Skyline-daily v21 by dividing the peak intensities of the native analytes by the peak intensities of the labeled standards (d4-hydrocortisone was used as a labeled standard for corticosterone) and multiplying by the inverse of the injection amount (in mL). All analytes are expressed in pg/mL in plasma, except for corticosterone and AA, which are expressed in ng/mL.

### 4.4. Statistical Methods

Fatty acid data were analyzed by two-way ANOVA using GraphPad Prism 8.3.1 (GraphPad software, San Diego, CA, USA), with maternal and postnatal diet as the factors, and with data separated for males and females. Post hoc comparisons were conducted with Tukey’s post hoc test. Serum concentrations of endocannabinoids were analyzed by univariate ANOVA. Partial eta-squared (ηp2) effect sizes were calculated and reported, with the alpha level of the tests set to 0.05. Bivariate correlations (Pearson’s coefficient correlations) examined the correlations between the endocannabinoids. Data are presented as the mean ± standard error of the mean (SEM). Significance was determined with *p*-values < 0.05.

## 5. Conclusions

In conclusion, intake of a diet high in LA during pregnancy and lactation alters SFA, MUFA, and PUFA composition in adult offspring’s brains, as well as endocannabinoids and testosterone in plasma. A maternal HLA diet increases the inflammatory mediator AA, and a postnatal HLA diet decreases the anti-inflammatory mediators DHA/n-6 DPA in a sex-specific manner in offspring’s brains. Furthermore, a maternal diet high in LA increases brain plasmalogen in male adult offspring, but not in female offspring. These findings suggest that the brains of the male offspring might be modified early in life due to the exposure to a diet high in LA. The exact mechanisms of this sex-specific variance, as well as the behavioral consequences of these changes, warrant further investigation. 

## Figures and Tables

**Table 1:** Effects of maternal and postnatal high-LA diets on fatty acid composition in the brains of 6-month-old male offspring.

	LLA Maternal Diet		HLA Maternal Diet		Two-Way ANOVA		
	LLA PN Diet	HLA PN Diet	LLA PN Diet	HLA PN Diet	Pmaternal	Ppostnatal	Pint
C14:0 Myristate	0.077 ± 0.005	0.106 ± 0.010	0.121 ± 0.021	0.083 ± 0.005	ns	ns	0.0095
C15:0 Pentadecanoate	0.023 ± 0.004	0.026 ± 0.005	0.032 ± 0.005	0.025 ± 0.003	ns	ns	ns
C16:0 Dimethylacetal	5.570 ± 0.507	5.468 ± 0.155	3.824 ± 0.110	4.478 ± 0.252	0.001	ns	ns
C16:0 Palmitate	21.69 ± 0.927	23.18 ± 0.927	23.47 ± 0.217	23.63 ± 0.153	ns	ns	ns
C16:1n7 Palmitoleate	0.171 ± 0.007	0.209 ± 0.013	0.208 ± 0.013	0.184 ± 0.017	ns	ns	0.0239
C17:0 Heptadecanoate	0.047 ± 0.005	0.060 ± 0.007	0.065 ± 0.009	0.056 ± 0.006	ns	ns	ns
C18:0 Dimethylacetal	7.840 ± 0.848	8.705 ± 1.238	4.611 ± 0.536	6.193 ± 0.318	0.0022	ns	ns
C18:0 Stearate	20.51 ± 0.336	20.24 ± 0.323	20.72 ± 0.373	21.34 ± 0.282	ns	ns	ns
C18:1n9 Oleate	14.26 ± 0.443	12.57 ± 0.087	14.90 ± 0.170	14.02 ± 0.431	0.0042	0.0008	ns
C18:1 Vaccenate	3.169 ± 0.065	2.977 ± 0.046	3.447 ± 0.103	3.282 ± 0.093	0.001	0.0251	ns
C18:2n6 Linoleate	0.211 ± 0.013	0.254 ± 0.017	0.303 ± 0.034	0.330 ± 0.026	0.0017	ns	ns
C20:0 Arachidate	0.078 ± 0.005	0.105 ± 0.015	0.105 ± 0.013	0.113 ± 0.014	ns	ns	ns
C18:3n6 Gamma Linolenate	0.016 ± 0.005	0.016 ± 0.004	0.015 ± 0.002	0.013 ± 0.003	ns	ns	ns
C20:1n9 Eicosenoate	0.436 ± 0.018	0.408 ± 0.020	0.597 ± 0.056	0.472 ± 0.041	0.0068	ns	ns
C20: 2n6 Eicosadienoate	0.046 ± 0.008	0.059 ± 0.005	0.069 ± 0.011	0.059 ± 0.006	ns	ns	ns
C22:0 Behenate	0.126 ± 0.028	0.074 ± 0.010	0.122 ± 0.011	0.109 ± 0.011	ns	ns	ns
C20:3 Homogamma Linolenate	0.142 ± 0.009	0.157 ± 0.012	0.183 ± 0.016	0.165 ± 0.018	ns	ns	ns
C22:1 Erucate	0.053 ± 0.009	0.045 ± 0.003	0.069 ± 0.008	0.056 ± 0.010	ns	ns	ns
C20:4n6 Arachidonate	7.985 ± 0.079	8.063 ± 0.082	8.610 ± 0.177	8.883 ± 0.178	<0.0001	ns	ns
C24:0 Lignocerate	0.193 ± 0.024	0.156 ± 0.010	0.242 ± 0.037	0.216 ± 0.018	0.0371	ns	ns
C20:5n3 Eicosapentaenoate	0.010 ± 0.002	0.021 ± 0.007	0.019 ± 0.004	0.006 ± 0.001	ns	ns	0.0095
C24:1n9 Nervoniate	0.094 ± 0.023	0.069 ± 0.008	0.139 ± 0.009	0.097 ± 0.017	0.0334	ns	ns
C22:4n6 Adrenate	2.705 ± 0.063	2.877 ± 0.186	3.157 ± 0.317	2.988 ± 0.196	ns	ns	ns
C22:5n6 Docosapentaenoate	0.281 ± 0.022	0.650 ± 0.107	0.372 ± 0.025	0.687 ± 0.033	ns	<0.0001	ns
C22:5n3 Docosapentaenoate	0.116 ± 0.012	0.165 ± 0.019	0.154 ± 0.023	0.164 ± 0.005	ns	ns	ns
C22:6n3 Docosahexaenoate	12.64 ± 0.807	12.99 ± 0.475	13.95 ± 0.284	12.62 ± 0.216	ns	ns	ns

Data are presented as the mean ± standard error of the mean (SEM). Two-way ANOVA was performed for statistical analysis with maternal diet and postnatal diet as two factors; *n* = 6–8. LLA: low linoleic acid; HLA: high linoleic acid; PN: postnatal; ns: not significant. P refers to the probability of a maternal, postnatal, or interactive effect.

**Table 2:** Effects of maternal and postnatal high-LA diets on fatty acid composition in the brains of 6-month-old female offspring.

	LLA Maternal Diet		HLA Maternal Diet		Two-Way ANOVA		
	LLA PN Diet	HLA PN Diet	LLA PN Diet	HLA PN Diet	Pmaternal	Ppostnatal	Pint
C14:0 Myristate	0.144 ± 0.022	0.114 ± 0.013	0.144 ± 0.008	0.123 ± 0.012	ns	ns	ns
C15:0 Pentadecanoate	0.034 ± 0.004	0.033 ± 0.005	0.041 ± 0.007	0.029 ± 0.005	ns	ns	ns
C16:0 Dimethylacetal	3.668 ± 0.491	4.778 ± 0.233	3.993 ± 0.394	4.346 ± 0.338	ns	ns	ns
C16:0 Palmitate	22.91 ± 0.508	23.57 ± 0.710	23.57 ± 0.402	22.69 ± 0.311	ns	ns	ns
C16:1n7 Palmitoleate	0.247 ± 0.026	0.213 ± 0.013	0.231 ± 0.012	0.222 ± 0.012	ns	ns	ns
C17:0 Heptadecanoate	0.073 ± 0.008	0.071 ± 0.007	0.072 ± 0.005	0.064 ± 0.006	ns	ns	ns
C18:0 Dimethylacetal	4.494 ± 0.746	5.887 ± 0.697	4.956 ± 0.219	5.043 ± 0.463	ns	ns	ns
C18:0 Stearate	20.10 ± 0.467	20.78 ± 0.201	20.54 ± 0.252	21.19 ± 0.190	ns	0.0409	ns
C18:1n9 Oleate	15.65 ± 0.805	13.36 ± 0.661	14.02 ± 0.320	14.80 ± 0.158	ns	ns	0.0221
C18:1 Vaccenate	3.479 ± 0.112	3.235 ± 0.099	3.319 ± 0.086	3.418 ± 0.051	ns	ns	ns
C18:2n6 Linoleate	0.295 ± 0.044	0.292 ± 0.009	0.264 ± 0.009	0.292 ± 0.014	ns	ns	ns
C20:0 Arachidate	0.138 ± 0.014	0.087 ± 0.005	0.103 ± 0.006	0.116 ± 0.007	ns	ns	0.003
C18:3n6 Gamma Linolenate	0.013 ± 0.002	0.013 ± 0.003	0.016 ± 0.003	0.009 ± 0.001	ns	ns	ns
C20:1n9 Eicosenoate	0.532 ± 0.043	0.506 ± 0.047	0.483 ± 0.050	0.592 ± 0.027	ns	ns	ns
C20: 2n6 Eicosadienoate	0.079 ± 0.013	0.064 ± 0.007	0.037 ± 0.005	0.056 ± 0.004	0.0083	ns	ns
C22:0 Behenate	0.127 ± 0.013	0.114 ± 0.018	0.104 ± 0.012	0.119 ± 0.007	ns	ns	ns
C20:3 Homogamma Linolenate	0.212 ± 0.024	0.218 ± 0.019	0.237 ± 0.016	0.199 ± 0.010	ns	ns	ns
C22:1 Erucate	0.072 ± 0.012	0.068 ± 0.007	0.062 ± 0.008	0.077 ± 0.010	ns	ns	ns
C20:4n6 Arachidonate	8.135 ± 0.185	8.150 ± 0.117	8.374 ± 0.281	8.378 ± 0.187	ns	ns	ns
C24:0 Lignocerate	0.221 ± 0.017	0.205 ± 0.012	0.216 ± 0.024	0.235 ± 0.017	ns	ns	ns
C20:5n3 Eicosapentaenoate	0.019 ± 0.005	0.012 ± 0.002	0.013 ± 0.002	0.014 ± 0.001	ns	ns	ns
C24:1n9 Nervoniate	0.127 ± 0.021	0.096 ± 0.008	0.117 ± 0.012	0.122 ± 0.009	ns	ns	ns
C22:4n6 Adrenate	3.047 ± 0.207	3.094 ± 0.126	3.287 ± 0.118	3.356 ± 0.156	ns	ns	ns
C22:5n6 Docosapentaenoate	0.349 ± 0.046	0.568 ± 0.017	0.299 ± 0.020	0.621 ± 0.055	ns	<0.0001	ns
C22:5n3 Docosapentaenoate	0.146 ± 0.021	0.173 ± 0.027	0.199 ± 0.015	0.168 ± 0.021	ns	ns	ns
C22:6n3 Docosahexaenoate	14.20 ± 0.601	14.23 ± 0.669	16.22 ± 0.823	13.99 ± 0.375	ns	ns	ns

Data are presented as the mean ± standard error of the mean (SEM). Two-way ANOVA was performed for statistical analysis with maternal diet and postnatal diet as two factors; *n* = 6–8. LLA: low linoleic acid; HLA: high linoleic acid; PN: postnatal; ns: not significant. P refers to the probability of a maternal, postnatal, or interactive effect.

## Data Availability

The raw data supporting the conclusions of this article will be made available by the authors on request.
